# Effects of Canned Pineapple Consumption on Nutritional Status, Immunomodulation, and Physical Health of Selected School Children

**DOI:** 10.1155/2014/861659

**Published:** 2014-11-20

**Authors:** Mavil May C. Cervo, Luisito O. Llido, Erniel B. Barrios, Leonora N. Panlasigui

**Affiliations:** ^1^School of Nutrition, Philippine Women's University, 1004 Manila, Philippines; ^2^Clinical Nutrition Services, St. Luke's Medical Center, 1102 Quezon City, Philippines; ^3^School of Statistics, University of the Philippines-Diliman, 1101 Quezon City, Philippines

## Abstract

This randomized, controlled trial examined the effects of canned pineapple consumption on immunomodulation, nutritional status, and physical health of ninety-eight (98) school children with mean age of 8.44 ± 0.20. The study participants were divided into three groups: Group A (33) includes subjects who were not given canned pineapple, Group B (33) includes those who were given 140 g, and Group C (32) includes those given 280 g of canned pineapple for nine weeks. Each major group was further divided into two groups: normal (N) and underweight (U) based on 2007 WHO Growth Reference Standards. Sociodemographic, anthropometric, physical examination, dietary intake, hemoglobin level, and immunological data were analyzed. Results showed a decrease in incidence of viral and bacterial infections for both Group B and Group C (normal and underweight) after canned pineapple consumption. Granulocyte production increased by 0.77–26.61% for normal weight subjects and 14.95–34.55% for underweight. CD16+56 count augmented by 20.44–22.13% for normal weight and 3.57–15.89% for underweight subjects. Thus, intake of both one can (140 g) and two cans (280 g) of canned pineapple may shorten the duration and incidence of infection and may increase the production of granulocytes and CD16+56, but intake of two cans (280 g) demonstrated higher granulocyte and CD16+56 production. This trial is registered with Philippine Health Research Registry: PHRR140826-000225.

## 1. Introduction

Despite the various improvements in the treatment of perpetually evolving viral and bacterial infections, they are still considered as significant problems in health care today, especially for developing countries like the Philippines. According to UNICEF (2012), most child deaths result from one of the following five causes or combination of acute respiratory infections, diarrhea, measles, malaria, and malnutrition. Pneumonia continues to be one of the leading causes of death among children not only in the Philippines, but also around the world. Of the estimated 6.9 M child deaths each year, 18% or 1.2 M are due to pneumonia and 11% or 750,000 are due to diarrhea, highly concentrated in the poorest regions and countries [1]. Mortality due to childhood pneumonia and diarrhea is strongly linked to poverty-related factors like malnutrition as well as viral and bacterial infections.

Based on the report submitted by the National Statistical Coordination Board (NSCB) in 2010, 5.97% or 5970 of 5–9-year-old children died due to pneumonia in 2009 in the Philippines. It is also the second leading cause of mortality in 5–14-year-old children and the leading cause of death in 1–4-year-old children. Among the top 10 leading causes of death in children in the country, five of these are caused by viral and bacterial infections [2]. The incidence of these infections is influenced by the efficiency of both the innate and the adaptive immune system of individuals and their interactions. Thus, it is of great importance to examine and utilize factors that may strengthen the immune system in order to reduce the incidence of viral and bacterial infections and hence the overall mortality rates.

Among the known factors that influence the strength of the immune system is malnutrition. The relationship between macro- and micronutrient deficiencies and immunity has been clearly established. It has been observed that malnutrition appears to lead to increased infection in some instances and increased resistance to infection in others [3]. Growing evidence suggests that eating the recommended amount of fruits and vegetables protects against a number of diseases. The potential benefits of increased fruit and vegetable intake may have been contributed by the antioxidant and phytochemical content of these food groups.

Although several studies have been done on various fruits and immunity, the pineapple (*Ananas comosus*), the country's top produce, has not been examined in children. Most of the studies conducted on pineapple and immunity used bromelain, which is often extracted in pineapple's stem. Also, most of these studies were done in vitro or in mice. Pineapple contains several phytochemicals like coumaric acid, ferulic acid, chlorogenic acid, and ellagic acid, as well as micronutrients that have established influences such as vitamin C, manganese, thiamin, riboflavin, pyridoxine, copper, and dietary fiber. Regular intake of pineapple may increase consumption of these phytochemicals and micronutrients and potentially influence some immunological markers and help improve the child's physical health. Hence, consumption of pineapple may have potential benefits in the alleviation of viral and bacterial infections in the country which may lead to decrease prevalence of death among children. In lieu, this research was done to determine the effect of nine-week canned pineapple consumption on various immune markers, nutritional status, and physical health of selected school children.

## 2. Materials and Methods

### 2.1. Experimental Design (See Figure 1)

This randomized, controlled trial involved 98 subjects which were divided into three major groups. Group A served as the control group or those subjects who were not given canned pineapple and maintained their usual intake. Group B served as the first treatment or those subjects who were given 140 g of canned pineapple, while Group C is the second treatment or those who were given 280 g of canned pineapple for nine weeks. Each major group was further divided into two subgroups: normal (N) and underweight (U) based on 2007 WHO Growth Reference Standards. Anthropometric parameters (weight and height), energy and nutrient intake, immunological markers (granulocytes, monocytes, leukocytes, lymphocytes, CD4, CD8, CD16+56, CD19, and CD20), hemoglobin, and incidence and duration of infection or illness were analyzed in the study.

### 2.2. Study Site Selection

The study was conducted in an elementary school (Fernando Ma. Guerrero Elementary School) in Paco, Manila, Philippines, with the highest number of students and the most number of students who are underweight based on the report submitted to the Department of Education-Division of Manila.

### 2.3. Subjects

A total of 98 students with mean age of 8.44 ± 0.20, divided randomly into three groups (A, B, and C), were included in the study. Each major group was further divided into two groups: normal (N) and underweight (U) based on 2007 WHO Growth Reference Standards.

The inclusion criteria were normal and underweight school children at the ages of 6–12 years old, having parents' or guardian's consent, having no known allergy to pineapple, being not included in the school's feeding program, and having no history of pineapple consumption for the past eight weeks. Subjects who fit the following criteria were excluded in the study: severely underweight, overweight, and obese children; menstruating; with previous history of any vitamin or mineral supplements or any pharmacotherapy within the past 2 months; having an acute or chronic infection or wound either at present or up to 2 weeks before the start of the study; having vaccination within 4 weeks before the start of the study or having any chronic or inflammatory disease; having undergone surgery; and any subject who will require hospitalization and aggressive treatment or medications.

#### 2.3.1. Selection Process

Subjects underwent extensive selection process to ensure that they satisfy the set inclusion criteria. All students aged 6–12 from the identified elementary school were given a pretested and validated survey form to assess whether they fit the set inclusion criteria. The survey form was verified using a one-on-one interview with the subjects and their parents. Those who meet the criteria (256) were then subjected to anthropometric assessment (weight and height). Physical examination was also done to eliminate subjects who have infection or any form of disease. Out of 256, only 99 students satisfy the set inclusion criteria. Potential participants were oriented with their parents or guardians regarding the details of the study. Parents or guardians of amenable subjects signed a volunteer consent form that was patterned after the ethical research guidelines by the Institutional Ethics Review Committee. Parents were also asked to fill up the 3-day food record (two nonconsecutive weekdays and one weekend). Ninety-nine students were then divided into three groups. Each group has 33 students at first. However, one student from Group CN dropped out of the study because she stopped going to school. These three groups were further divided into two groups: those who are normal and those who are underweight. For each major group, twenty-three are classified as normal and 10 of which are classified as underweight.

### 2.4. Test Food

Group B was given 140 g (1 can) of canned pineapple (pineapple tidbits), while Group C was given 280 g (2 cans) of canned pineapple every day for nine weeks. The test food was produced by Del Monte plantation and cannery in Mindanao, Philippines.

### 2.5. Feeding Intervention

The intervention period (feeding) lasted for nine weeks. The control (Group A) was not given any intervention thus maintaining their usual dietary intake. Experimental (Groups B and C) was instructed to consume 140 g of canned pineapple or 280 g of canned pineapple, respectively, a day for nine weeks while maintaining their usual dietary intake. The test food was prepared in the school's canteen and delivered to all participants under the experimental group every morning. In order to ensure that the participants really consumed the test food, the subjects' respective teachers were asked to monitor their students. The researcher and helpers collect the containers and weigh if there are any leftovers. During weekends, all subjects under the treatment group were required to go to school to consume their test foods.

### 2.6. Data Collection and Analysis

Subjects under the experimental group were fed with canned pineapple every day for nine weeks. The participants were monitored and assessed using different parameters.

Medical history and family history of the subjects were taken before the start of the study to determine allergies or other diseases. Presence of blood coagulation problems is ruled out. Sociodemographic profile of the subjects was taken before the start of the study.

Anthropometric data include weight (measured using Platform balance, Detecto Eye Level Physician Scale, MO, USA) and height (using Microtoise, Tanita Corporation, Tokyo, Japan). Anthropometric data were gathered before and at the 4th and 9th weeks of study intervention.

Usual dietary intake was assessed using three-day food record (FR). Food record was used to quantify the actual food intake of the subjects during the study. This was taken before (baseline), 4th week (test 1), and 9th week (final) of study intervention. Each record includes two nonconsecutive weekdays and one weekend. FR data was validated through interview. The nutrient intake of the subjects was computed using the Philippine Food Composition Tables (FNRI-DOST, 1997) and Food Exchange Lists for Meal Planning (FNRI-DOST, 1994).

To determine the incidence of viral and bacterial infections, physical examination was done every week. Laboratory tests measure levels of metabolites, particularly in blood, to evaluate the body's state of health or its response to various treatments. In this study, hemoglobin and immunological tests were done before and after the study. Immunological test was done to determine the effect of canned pineapple consumption on immune system. It provides data on immunological markers such as CD4, CD8, CD19, CD20, and CD16+56. It also includes data on leukocyte, lymphocyte, granulocyte, and monocyte count. Since the immunological test is quite expensive and due to the limited budget, only five subjects per group (total of 30 subjects) were included in the immunological analysis.

Physical examination and blood extraction were done in the school auditorium. Ten milliliters of blood was extracted from the antecubital vein for immunological analysis. Immune panel data from the subjects' blood samples was obtained using flow cytometry analysis performed on a Becton-Dickinson FACScan (Becton-Dickinson, Mountain view, CA, USA). Hemoglobin and immunological analyses were done at St. Luke's Medical Center, Quezon City.

Each subject was given a notebook that serves as their diary to record possible discomfort related to gastrointestinal effects such as diarrhea, abdominal distension, flatulence, nausea, or any allergic reaction. Entries were checked every day after delivery of test foods to individual classroom of subjects.

All members of the research team have the necessary background and skills needed for the study. They were oriented on the data collection techniques to ensure the uniformity of the methods that were employed. The medical technologists as well as medical doctors were blinded to the group assignments of the study participants to prevent bias. All forms were pretested and validated prior to the actual study. Laboratory equipment was calibrated and underwent usual quality control methods. Collected data was reviewed for completeness and accuracy by the principal investigator.

### 2.7. Statistical Analysis

Changes in different parameters were determined from baseline until the end of intervention. Anthropometric, dietary (from food record), and biochemical results were presented as mean ± standard error (SE). Determination of the significant differences was analyzed using paired sample *t*-test and analysis of covariance (ANCOVA) in repeated measures and using Duncan multiple-range tests (DMRT). Multiple-linear regression was used to determine the relationship of nutrients on different immune markers. The SAS version 9.1 data analysis software (SAS Institute Inc., NC, USA) was used in testing the significance among the data. Microsoft Office Excel 2010 (Microsoft Corporation, WA, USA) was used in processing the other numerical data gathered. The level of significance is set at *P* < 0.05. Results of physical examination and sociodemographic data were analyzed using descriptive statistics and were presented in frequencies and percentages.

## 3. Results 

### 3.1. Baseline Characteristics of Subjects (See Table 1)

Table 1 shows the baseline characteristics of subjects based on anthropometric measurements and immunological markers.

### 3.2. Immunological Markers

Table 2 shows the average increase in levels of innate immunological markers. It is evident that granulocyte count is significantly affected by baseline level (*P* < 0.0002) as well as the treatment (*P* < 0.0378). Granulocyte count increases as canned pineapple consumption increases. Among the normal weight subjects, Group AN (control) exhibited a reduction in granulocyte count by 14.19% and those who have taken one can of pineapple (Group BN) yielded an increase of 0.77%. Group C or those who consumed two cans of pineapple produced 26.61% more granulocytes during the study period, while underweight subjects produce more granulocytes with intake of canned pineapple. Group AU's granulocyte count significantly decreased by 5.67%, while Groups BU and CU escalated by 14.95% and 34.55%, respectively.

After nine weeks of study, white blood cells (WBCs) are affected by baseline level (*P* < 0.0068) but do not vary significantly across different treatments (*P* < 0.3282). Among the normal weight subjects, Group CN has the highest average increase in WBC count followed by Groups BN and AN, respectively. For those who are underweight, Group CU has the highest increase in WBC count followed by Groups BU and AU. Lymphocyte count is not affected by baseline level (*P* < 0.6734) and is not significantly affected by the treatment (*P* < 0.4622) as well. Group AN has the highest increase in level of lymphocytes followed by Group CN and Group BN among the normal subjects. Among underweight subjects, Group CU has the highest raise in lymphocyte count followed by Groups AU and BU, respectively.

Just like WBC, monocyte count is found to be affected by baseline level (*P* < 0.0011), but it was not significantly affected by the treatment (*P* < 0.8274). Among normal weight subjects, Group BN yielded the highest average increase followed by Groups AN and CN. For the underweight subjects, Group CU has the highest increase followed by Groups BU and AU.

Table 3 illustrates the average change in adaptive immunological markers. CD4 count is not affected by the baseline and treatment level at *P* < 0.3834 and *P* < 0.4241, respectively. Among normal subjects, Group AN has the highest average increase followed by Group CN, while CD4 count of BN decreased. Among underweight subjects, Group BU has the highest increase in CD4 count followed by Groups CU and AU.

Among subjects with normal weight, Group AN has the highest average increase in level of CD8 count followed by Group BN and Group CN, while, for those who are underweight, Group AU has the highest increase in CD8 count followed by Groups BU and CU. However, using the analysis of covariance, CD8 count is found to be not significantly affected by baseline level (*P* < 0.4202) and by treatment (*P* < 0.5082).

CD20 count for the normal weight groups diminished during the course of the study. Group CN has the highest average decrease followed by Groups BN and AN. Among underweight subjects, Group CU has the highest average increase followed by Group BU, while Group AU's CD20 count declined after nine weeks of study intervention. Though ANCOVA, the results were not affected by baseline or treatment at *P* < 0.1516 and *P* < 0.7786.

Though CD19 count was affected by baseline level (*P* < 0.0426) it was not significantly affected by the treatment (*P* < 0.8332). Like CD20, CD19 count of each group with normal weight decreased. Group CN has the highest average decrease in level of CD20 count followed by Groups BN and AN, respectively. Among underweight subjects, only Group AU's CD19 count increased, while those of Groups BU and CU diminished.

Among the CD markers, only CD16+56 count is significantly affected by both baseline and treatment at *P* < 0.0238 and *P* < 0.0386. Among the subjects with normal weight, Group CN's CD16+56 count augmented with an average of 22.13% followed by Group BN with 20.44%, while Group AN decreased with an average of 19.93%. For those who are underweight, Group CU's CD16+56 count escalated by 15.89% followed by BU with 3.57%. Just like with the normal weight subjects, Group AU declined by 2.17%. These data show that intake of canned pineapple can increase CD16+56 count by 20.44 to 22.13% for those who have normal weight and 3.57 to 15.89% for those who are underweight.

### 3.3. Nutrients and Immunological Markers (See Table 4)

Based on the results of the linear regression analysis, as shown in Table 4, among the nutrients studied, only vitamin C was found to be associated with granulocyte count and white blood cell count. Results indicate that every 1 mg increase in vitamin C intake will result in an increase of 86.75 cells/mL increase in granulocyte count and an increase of 103.45 cells/mL in WBCs. In terms of CD16+56, only iron was found to be associated. In every 1 mg increase in iron intake, there will be 68.61 cells/mL increase in CD16+56.

### 3.4. Hemoglobin

Level of hemoglobin had increased for both normal and underweight subjects. Among normal subjects, Group CN has the highest increase by 0.60 g/dL followed by Groups BN (0.59 g/dL) and AN (0.30 g/dL), respectively, while for those underweight subjects, Group CU has the highest increase by 0.57 g/dL followed by Groups BU and AU with 0.40 g/dL and 0.19 g/dL, respectively. All data are found to be statistically significant except for Groups AU and BU (Table 5).

### 3.5. Incidence and Duration of Infection (see Table 6)

Physical examination of normal weight subjects shows that compared to control (Group AN), Groups BN and CN had zero incidence of infection at week 1 and had much lower incidences during the succeeding weeks which even became zero during the last week of the study intervention. The same thing goes for those subjects who are underweight. Group AU has the highest incidence of infection starting from week 1 till the final week of study intervention compared with Groups BU and CU. It is also noteworthy that the incidence of infection is high during the second, third, and fifth weeks of study intervention. This is probably due to occurrence of typhoons and climate change.

Aside from the incidence of infection, the duration or extent of an infection was analyzed for those who had an infection. For normal weight subjects, Group AN's average length or duration of infection lasted for 4–8 days with an average of 5.28 or 5 days. Groups BN and CN lasted for 3-4 days with an average of 3.26 and 3.30 days (3 days), respectively. For those who are underweight, Group AU's average duration of infection lasted for 4–8 days with an average of 5.62 or 5-6 days, while both Groups BU and CU lasted for 3–5 days with an average of 4.1 (4 days) and 3.5 (3-4 days), respectively. These data only reveal that the average duration of infection is much shorter for subjects who consumed canned pineapple compared with the control.

## 4. Discussion

Pineapple is a multiple fruit that is truly abundant in the Philippines. It is a good source of many nutrients such as vitamins B2 (riboflavin), C (ascorbic acid), manganese, and phytochemicals [4]. Consumption of pineapple and its components has been linked with immunity [5–18]. Some researchers also associated pineapple with cancer [19–23], wound healing [24–26], diabetes mellitus and liver disorders [27–31], diarrhea or other disorders of the gastrointestinal tract [32, 33], and even tuberculosis [34].

Microbial agents are encountered throughout life. Young children have an average of six to seven colds per year, but 10–15% of children have at least 12 infections per year [35]. In this study, incidence of viral and bacterial infections on both normal and undernourished children increased tremendously during the second, third, and fifth weeks of study intervention which may be associated with the occurrence of typhoons and climate change. Groups B and C, both normal and underweight, had zero incidence of infection at week 1 and have much lower incidences during the succeeding weeks which even became zero during the last week of the study intervention. Aside from the incidence of infection, the duration or extent of an infection was analyzed for those who had an infection. Data reveal that the average duration of infection is much shorter for subjects who consumed canned pineapple compared with the control. In addition, recovery also took much longer duration for underweight subjects than those who are normal in weight. Subjects mostly suffered from common colds, cough, fever, and upper respiratory tract infection during the study.

The human immune system has a central role in protecting against various infectious agents such as bacteria and viruses. The essential features of the immune system are an innate component that functions as a first-line of defense and an adaptive component that takes longer to mobilize but confers specificity and exhibits memory. Unlike adaptive immune system, which may take days to mobilize, innate system is extremely quickly mobilized [36].

Among subjects with normal weight, Group CN has the highest average increase in levels of immunological markers such as granulocytes and CD16+56. Group CN has four times higher on the level of granulocytes than Group AN (control). For the normal weight subjects, Group AN exhibited reduction in granulocyte count by 14.19%. Group BN yielded an increase of 0.77% and Group CN produced 26.6% more granulocytes during the study period. For the underweight subjects, Group AU's granulocyte count decreased by 5.67%, while Groups BU and CU increased by 14.95% and 34.55%, respectively. Findings suggest that canned pineapple can possibly be a booster in the production of granulocytes in both normal and underweight subjects. By consuming canned pineapple, normal weight subjects can boost their granulocyte count by 0.77–26.61% and 14.95–34.55% for those who are underweight. It also demonstrates that canned pineapple has a more pronounced effect on granulocyte production of underweight subjects compared with those who are normal in weight. Granulocytes play an important role in the innate immune system. It has a phagocytic capacity in destroying or killing viruses and bacteria and is also important in intracellular communication [37]. Granulocytes include neutrophils, eosinophils, and basophils.

In terms of CD16+56, Group AN showed a decline by 19.93%, while Groups BN and CN produced more CD16+56 by 20.44% and 22.13% correspondingly. For the underweight subjects, Group AU's CD16+56 count also declined by 2.17%, while Group BU and CU augmented by 3.57% and 15.89% respectively. Based on the results, it is also apparent that increasing consumption of canned pineapple can increase CD16+56 count for both normal and underweight subjects. CD16+56 mediates antibody-dependent cytotoxicity of foreign cells, phagocytosis, and other antibody-dependent responses and is also involved in the activation pathways of natural killer cells [36]. Though level of CD16+56 escalated on both Groups B and C (both normal and underweight subjects), it is evident that it is still within the normal range (212–612 cells/mL); thus, the increase in amount may not lead to autoimmunity.

Among the immunological parameters, only granulocytes and CD16+56 count are found to be statistically significant. This suggests that canned pineapple consumption has distinct effect on both innate and adaptive immune system. Though there is no significant effect on CD4 and CD8, which are often used in many studies, the significant increase in CD16+56 demonstrates the effect of canned pineapple consumption on the adaptive immune system. One important feature of both granulocytes and CD16+56 is their capacity to express natural killer cells which are important in killing certain tumor cells and are cytotoxic for both viruses and bacteria. Natural killer cells have receptor and inhibitory receptor and thus can selectively kill virus-infected and transformed cells while sparing normal cells [37].

In addition, it is best to remember that the innate and adaptive immune systems are interconnected and overlapping cells. The adaptive immune system is usually triggered by the innate immune system and only comes into play if the innate immune system fails to overwhelm the microorganism or the microorganism has found a way to avoid interaction with the innate system [38]. This means that, without the innate immune system, the adaptive immune system will not be able to do its job efficiently for the adaptive immune system depend on prior activation and participation of the innate immune system. As shown in this study, canned pineapple consumption was able to significantly increase the production of granulocytes which is an important marker of the innate immune system as well as CD16+56 which, on the other hand, is a marker of the adaptive immune system.

Hemoglobin level increased for both normal and underweight subjects especially to those who consumed canned pineapple. Before the study commenced, 20% of the subjects from Group BU had a hemoglobin level of less than 10 g/dL and therefore suffered from anemia. After nine weeks of canned pineapple consumption, their hemoglobin level became normal. The vitamin C content of the canned pineapple may be somehow responsible for this positive effect since vitamin C enhances iron absorption. Moreover, the increase in CD16+56 can also be a factor since this marker is essential in regulation of hematopoiesis.

The problem of disease and infection is multifaceted. Malnutrition, including both calorie and micronutrient deprivation, contributes to reduced immunity and increases the prevalence of infection. Infection and disease develop when the host's immune system is insufficient to entirely protect against potential pathogens. Once an infection is established, an array of immune responses is set into action to control the disease and avert it from reappearing. Based on the anthropometric data, there is a slight increase in height and weight among groups, which is expected since canned pineapple can only provide 76–152 kcal for one and two cans.

Food record data show that intake of subjects did not vary much all throughout the study period which also indicates that the improvement in the different parameters particularly on granulocyte and CD16+56 count can be attributed to the canned pineapple. Also, intakes of canned pineapple have contributed a marked increase in almost all nutrients especially riboflavin, iron, and vitamin C intakes among normal weight and underweight subjects when compared with baseline data. Forty-three percent of the subjects' mean vitamin C intake comes from canned pineapple (140 g) alone. However, most of the groups have inadequate consumption of calcium, iron, vitamin A, vitamin B1, and vitamin C throughout the study. This data reflects micronutrient deficiencies that may have direct effects on immunity and susceptibility to disease. These nutrients, with the exception of vitamin C, iron, and calcium, are not abundantly found in canned pineapples and thus inadequate intakes are not affected by the study. Moreover, the subjects' intake was already inadequate in calcium, iron, and vitamins A and C prior to canned pineapple supplementation. These micronutrients play an important role in immune system modulation. Vitamin A, for instance, is essential in the integrity of the immune system. All lymphocytes require vitamin A to develop and function properly. Consequently, vitamin A deficiency can alter the response of some antibodies to antigens and may also exert effects on the network of cytokines secreted during an immune response. Vitamin C is important in the biosynthesis of collagen and therefore promotes wound healing and also protects the skin. Furthermore, just like vitamin A, it is also an important antioxidant which protects the body from free radicals. Iron is essential for the normal function of the immune system. Since calcium is required in cellular movement, it is believed to have a vital role in increasing the body's resistance to infections. This inadequacy in micronutrients may somehow affect the results of the study particularly on the immune markers.

Among the nutrients studied, only vitamin C was found to be associated with granulocyte count and white blood cell count. Every 1 mg increase in vitamin C intake may lead to 86.75 cells/mL increase in granulocyte count and 103.45 cells/mL increase in WBCs. This result coincides with the findings of de la Fuente et al. [39] when administration of 1 g vitamin C to 30 subjects for 16 weeks increased the lymphoproliferative capacity and phagocytic function of neutrophils, which is a type of granulocytes. In terms of CD16+56, only iron was found to be associated (*P* < 0.0080). In every 1 mg increase in iron intake, there will be 68.61 cells/mL increase in CD16+56. This can also be due to the role of iron in normal functioning of the immune system.

Aside from the dietary intake and nutritional status of children, social and environmental influences have a major impact on the health and well-being of children. Family income is central to the health and well-being of children. Based on the sociodemographic form filled up by the subjects' parents, the mean monthly income is Php 6,434.00 with 43.3% of them having Php 2,500.00. With a minimum average of six members per household, Php 29.47 is spent for food per person daily. This means that some of these children have less access to nutritious foods which leads to poor health. This may also be the reason why subjects have inadequate intake of many micronutrients. Environmental factors such as living conditions as well as weather/climate may have affected the results of the study. Colds are common during rainy days because of the seasonal increase in viral infections particularly rhinovirus. The highest incidence of rhinovirus infection or common colds occurs from August to October. This can also be a factor why there is a remarkable increase in incidence of infection during the 2nd, 3rd, and 5th physical examinations.

Based on the results of the study, it can be concluded that canned pineapple consumption can lower the incidence of viral and bacterial infections and further increase the production of granulocytes by 0.77–26.61% for normal weight subjects and 14.95–34.55% for those who are underweight. It can also boost the production of CD16+56 by 20.44–22.13% for normal weight subjects and 3.57–15.89% for underweight subjects. Both one can (140 g) and two cans (280 g) of canned pineapple may increase the production of granulocytes and CD16+56, but two cans of (280 g) pineapple consumption demonstrated higher granulocyte and CD16+56 production.

The time of the study might not be ideal due to occurrences of typhoons. Furthermore, colds are common during rainy days because of seasonal increase in viral infection. For future studies, the researchers recommend to conduct the study during both rainy and dry seasons. More subjects should be recruited in the study and the intervention period should be much longer to see more significant results. Research using both fresh and canned pineapple fruit is recommended. Further studies on the effect and mechanism of pineapple and its component on nutritional status and immunology are also warranted.

## Figures and Tables

**Figure 1:**
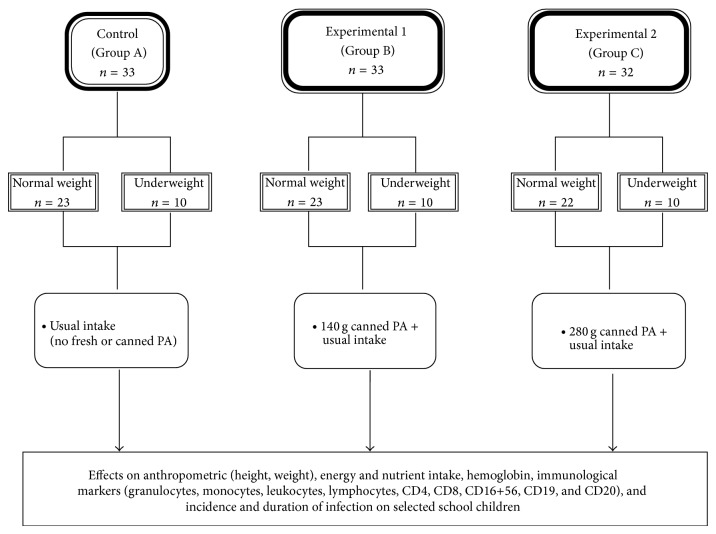
Experimental design (*n* = 98).

**Table 1:** Baseline characteristics of subjects.

Parameters	AN		BN		CN		AU		BU		CU	
	Mean	SEM	Mean	SEM	Mean	SEM	Mean	SEM	Mean	SEM	Mean	SEM
Height (cm)	119.92	2.08	126.36	2.87	128.33	2.72	114.55	3.13	115.40	2.42	118.28	2.73
Weight (kg)	22.78	1.21	27.75	2.33	27.81	1.64	18.36	1.41	18.80	0.96	19.35	1.04
Immunological markers												
WBC	6684.00	717.79	7508.00	716.45	7532.50	641.70	8015.00	728.13	7487.50	816.92	7577.50	873.11
Granulocytes	3347.00	510.13	3837.40	452.64	4121.75	285.08	3874.50	199.33	3952.00	657.58	4265.25	667.15
Lymphocytes	2748.00	340.59	3209.00	293.66	2884.25	344.97	3533.75	657.82	2926.50	154.88	2820.25	286.25
Monocytes	589.00	99.39	461.60	43.62	526.50	72.30	606.75	78.55	609.00	106.87	492.00	66.27
CD4	942.80	108.21	884.40	242.88	954.75	114.31	1189.25	255.82	956.25	62.67	989.00	121.30
CD8	716.00	60.86	1176.60	164.23	1036.25	180.35	1030.00	179.79	1055.75	67.78	865.25	168.02
CD16+56	444.46	66.65	386.60	128.67	385.00	54.27	531.00	63.29	462.00	135.16	440.00	52.45
CD20	639.80	75.00	647.20	60.97	490.75	67.58	697.50	138.03	597.25	60.92	602.75	139.94
CD19	641.40	75.60	635.80	66.05	481.50	74.01	769.00	199.55	595.50	65.69	601.75	132.05

**Table 2:** Mean change in levels of innate immunological markers among groups.

Group	Immunological markers (cells/mL)			
	Granulocytes^*^	WBC	Lymphocytes	Monocytes
Normal				
AN	−507.97	259.64	559.72	18.20
BN	34.50	373.92	191.98	52.41
CN	1315.34	1294.63	323.97	2.07
Underweight				
AU	−203.04	297.50	240.00	16.25
BU	669.34	890.00	234.75	28.50
CU	1707.97	1777.50	541.00	131.00

^*^Significant at *P* < 0.05.

**Table 3:** Mean change in levels of adaptive immunological markers among groups.

Group	Immunological markers (cells/mL)				
	CD4	CD8	CD16+56^*^	CD20	CD19
Normal					
AN	84.65	187.20	−76.30	−45.80	−53.80
BN	−110.87	66.91	99.48	−106.02	−97.92
CN	63.88	38.25	110.64	−119.06	−119.06
Underweight					
AU	32.00	127.25	−8.32	−65.50	102.37
BU	93.37	115.25	17.37	103.36	−99.50
CU	42.75	44.00	79.46	163.76	−75.00

^*^ *P* < 0.05.

**Table 4:** Interaction of selected nutrients and immune markers.

Dependent variable	Independent variable	Coefficient	Probability^*^
Granulocyte	Vitamin C	86.75	0.0269
WBC	Vitamin C	103.45	0.0264
CD16+56	Iron	68.61	0.0080

^*^ *P* < 0.05.

**Table 5:** Mean change in hemoglobin level among groups.

Group	Hg (g/dL)				*P* value^*^
	Baseline		Posttest	
	Mean	SEM	Mean	SEM
Normal					
AN	12.80	0.23	13.10	0.19	*P* = 0.023^*^
BN	12.76	0.20	13.34	0.19	*P* = 0.002^*^
CN	12.68	0.23	13.28	0.15	*P* = 0.008^*^
Underweight					
AU	12.95	0.52	13.14	0.35	*P* = 0.301
BU	11.77	0.20	12.17	1.05	*P* = 0.371
CU	12.76	0.33	13.33	0.29	*P* = 0.039^*^

^*^ *P* < 0.05.

**Table 6:** Incidence of infection among groups.

Group	Students with infection (%)								
	Week 1	Week 2	Week 3	Week 4	Week 5	Week 6	Week 7	Week 8	Week 9
Normal									
AN	8.70	30.43	34.78	13.04	21.74	13.04	13.04	13.04	4.35
BN	0.00	13.04	4.35	0.00	13.04	4.35	4.35	4.35	0.00
CN	0.00	9.09	4.54	4.54	9.09	9.09	0.00	0.00	0.00
Underweight									
AU	30.00	30.00	30.00	10.00	30.00	30.00	10.00	10.00	20.00
BU	10.00	20.00	10.00	0.00	10.00	10.00	0.00	0.00	0.00
CU	10.00	10.00	0.00	10.00	0.00	0.00	0.00	0.00	0.00
