# Corrigendum to “Effects of Canned Pineapple Consumption on Nutritional Status, Immunomodulation, and Physical Health of Selected School Children”

**DOI:** 10.1155/2019/5731952

**Published:** 2019-07-01

**Authors:** Mavil May C. Cervo, Luisito O. Llido, Erniel B. Barrios, Leonora N. Panlasigui

**Affiliations:** ^1^School of Nutrition, Philippine Women's University, 1004 Manila, Philippines; ^2^Clinical Nutrition Services, St. Luke's Medical Center, 1102 Quezon City, Philippines; ^3^School of Statistics, University of the Philippines-Diliman, 1101 Quezon City, Philippines

In the article titled “Effects of Canned Pineapple Consumption on Nutritional Status, Immunomodulation, and Physical Health of Selected School Children” [1], the Conflicts of Interest section should read as follows.

“The authors declare that Del Monte Philippines, Inc. (DMPI) provided the canned pineapple and financial support but had no role in the design and conduct of the study; collection, analysis, and interpretation of the data; preparation and review of the manuscript; and decision to publish. Dr. Panlasigui previously conducted studies for DMPI on a juice drink, ending in 2010.”
